# The role and mechanisms of exosome microRNA in regulating metastasis within the tumor microenvironment of prostate cancer

**DOI:** 10.3389/fonc.2025.1580314

**Published:** 2025-06-10

**Authors:** Yongcheng Song, Qinzhang Wang, Bin Liang, Songnian Zou

**Affiliations:** ^1^ Medical College, Shihezi University, Shihezi, China; ^2^ The First Affiliated Hospital of Shihezi University, Shihezi, China; ^3^ Department of Urology, Changzhou Cancer (Fourth People’s) Hospital, Changzhou, Jiangsu, China

**Keywords:** PCA, exosome microRNA, metastasis, tumor microenvironment, epithelialmesenchymal transition

## Abstract

Prostate cancer (PCa) metastasis remains a significant challenge in clinical treatment, resulting in limited effective treatment options and poor clinical outcomes. Recent studies have highlighted the important function of exosome microRNAs (miRNAs) in governing metastatic processes within the tumor microenvironment (TME). Our review examines the mechanisms by which exosomal miRNAs contribute to PCa metastasis, focusing on their involvement in regulating tumor invasion and migration, epithelial-mesenchymal transition, and modulating immune responses. The review also discusses the implications of these findings for therapeutic targeting of exosomal miRNAs, indicating that they may act as potential biomarkers for prognosis and therapeutic while offering novel avenues for treatment strategies aimed at inhibiting metastasis. By elucidating the intricate interplay between exosomal miRNAs and the TME, this review aims to providing new insights into PCa metastasis while offering a theoretical foundation for future clinical research.

## 1 Introduction

Prostate cancer (PCa) is one of the most common tumors and currently ranks as the second leading cause of cancer-related fatalities among men (1). With China’s shift towards an aging population, the annual incidence of PCa is steadily rising in the country (2). Early-stage PCa, typically characterized by localized tumors, can often be effectively treated through surgical resection, chemotherapy, radiotherapy, and hormone therapy (3). Unfortunately, around 30% of cases advance to metastatic disease, where androgen deprivation therapy (ADT) serves as the main treatment (4), but most patients ultimately develop life-threatening castration-resistant PCa (CRPC) (5).

Consequently, CRPC is classified as a hormone-independent malignancy, prompting increased research efforts to identify potential metastatic mechanisms for the development of targeted therapeutic strategies (6). Furthermore, most patients diagnosed with advanced PCa present with multiple metastases, which affect not only nearby lymph nodes but also distant sites such as the bones, liver, brain, and lungs (7, 8). Among these sites, bone metastasis is considered an incurable manifestation, significantly contributing to the disease’s distinct morbidity and mortality (9). Despite this, the molecular mechanisms driving the formation of metastases are still not well understood, resulting in a dearth of effective treatment options and unsatisfactory five-year survival rates.

Current treatment strategies and investigations into metastatic primarily concentrate on intrinsic tumor cells, often overlooking the tumor microenvironment (TME), which is significantly contribute to the progression of PCa (10). The TME is composed of tumor cells as well as a diverse array of other cell types, such as cancer-associated fibroblasts (CAFs), immune cells, endothelial cells, and stromal cells. Additionally, it encompasses extracellular matrix (ECM) proteins, cytokines, chemokines, and extracellular vesicles (EVs) (11). Inside the TME, these components interact through a complex network, and this crosstalk significantly influence the development and progression of CRPC (12). EVs play an essential role in facilitating communication between cells within the TME, with exosomes constituting the most prominent subgroup. In recent years, exosomes have garnered increasing recognition for their roles in the progression of CRPC (13). These nanoscale vesicles act as vehicles for transporting specific molecules, thereby facilitating intercellular information transfer. Exosomes are composed of diverse bioactive substances, such as nucleic acids, lipids, proteins, carbohydrates, and metabolites, with non-coding RNAs (ncRNAs), particularly microRNAs (miRNAs), being more enriched and stable in exosomes than in circulating ncRNAs due to their protection by a bilayer lipid membrane (14, 15). Research indicates that the miRNAs carried by these exosomes can regulate gene expression, thereby affecting multiple cellular processes and playing critical roles in cancer development (16). This review seeks to elucidate how exosome miRNAs influence the metastatic behavior of PCa by modulating the TME, exploring their potential as biomarkers and therapeutic targets, and providing new insights into PCa metastasis while offering a theoretical foundation for future clinical research.

## 2 Basic characteristics of exosomes and miRNAs

### 2.1 Exosomes

Exosomes are extracellular vesicles that measure between 30 and 150 nanometers in diameter, distinguished by their lipid bilayer membrane. he primary constituents of exosomes include nucleic acids, lipids, proteins, and other molecules, with their composition shaped by the source cells and the overall health of the organism (17). Proteins serve as the primary components of exosomes and significantly influence tumor invasion and migration abilities, thereby facilitating tumor progression and metastasis (18). Exosomes carry diverse nucleic acid components, including miRNAs, mRNAs, and lncRNAs. Large-scale profiling of exosomal cargo has revealed their molecular complexity: 4,563 unique proteins, 1,639 mRNAs, and 764 miRNAs are selectively packaged into these vesicles. These biomolecules functionally mediate intercellular communication through targeted molecular exchanges. miRNAs can transfer to target cells and modulate receptor cell signaling pathways by fusing with the target cell membrane (19). The composition of exosomes varies based on the tissues from which they are secreted. These components can influence target cells in various ways, enabling information transfer and participating in multiple physiological and pathological processes, including tumorigenesis, antigen presentation, vascular remodeling, drug resistance, and metastasis (20). Recent studies have shown that exosomes can function as both prognostic molecular markers and novel therapeutic targets for inhibiting the progression of PCa (21, 22).

### 2.2 miRNAs

miRNAs are a subclass of endogenous ncRNAs expressed in multicellular organisms, typically measuring about 22 nucleotides in length (23). miRNAs perform various physiological roles, primarily by regulating specific genes at the post-transcriptional level. Approximately 2,500 distinct miRNAs have been discovered in humans, and researchers generally believe that they regulate more than half of the genes involved in protein synthesis. The primary role of miRNAs in gene expression is their capacity to bind complementarily to the 3′-untranslated region (3′-UTR) of target mRNAs. This interaction leads to either the degradation of the target mRNA or the repression of its translation (24). Moreover, miRNAs can facilitate the transmission of genetic information between different cells and across tissues (25). Previous studies have demonstrated that miRNA expression differs between tumor cells and normal tissues, influencing tumor formation, growth, invasion, migration, and metastasis (26). They play crucial roles in fundamental cellular activities such as proliferation, differentiation, migration, apoptosis, and metabolism in nearly all types of cells (27). miRNAs exhibit stability in both tissues and biological fluids, rendering them suitable for established analytical methods. Given their essential regulatory roles in various biological pathways, researchers have considered miRNAs as potential prognosis biomarkers and therapeutic targets in numerous diseases, especially cancer.

## 3 The multiple mechanisms of TME driven PCa bone metastasis

The likelihood of distant metastasis in advanced PCa can reach as high as 70%, accompanied by a relatively poor prognosis (28). Investigating the malignant biological characteristics of PCa and the mechanisms underlying its bone metastasis is essential for identifying potential therapeutic strategies. Current research suggests that tumor metastasis is primarily facilitated through a complex cascade of processes, including the detachment of tumor cells from the original site, local invasion, and infiltration into the bloodstream, circulatory transport, extravasation, colonization of target organs, and significant growth (29). Theoretical frameworks that drive and facilitate this metastatic cascade include five main concepts: the clonal evolution theory, the epithelial-mesenchymal transition (EMT) theory, the seed and soil theory, the circulating tumor cells theory, and the TME theory.

### 3.1 EMT and matrix remodeling

EMT is an important phenotypic process underlying cancer metastasis, enabling malignant epithelial cells to transition to a mesenchymal phenotype. This transition significantly enhances their invasiveness and metastatic potential (30, 31). In PCa, the EMT process is intricately influenced by the TME and the complex interactions among various cellular components, such ad glandular epithelial and stromal cells (32). As PCa progresses, malignant epithelial cells undergo transformation facilitated by a reactive stroma, contributing to promoting tumor metastasis (12). A key contributor to this dynamic is CAFs, which are characterized by the expression of fibroblast activation protein. CAFs are often exhibit increased expression in cases of poorly differentiated PCa and bone metastases. CAFs not only facilitate the degradation of the basement membrane but also remodel the ECM by secreting ECM components and degrading enzymes (33). Notably, Liu et al. highlighted that CAFs could promote malignant phenotypes in PCa through ATG5-dependent autophagy (34).

Central to the regulation of EMT are transcriptional factors includes Zinc finger E-box-binding homeobox 1/2 (ZEB1/2), snail family transcriptional repressor 1 (SNAIL), snail family transcriptional repressor 2 (SNAI2), and twist family BHLH transcription factor 1 (TWIST1). These factors are modulated by various growth factor signaling pathways, particularly transforming growth factor-beta (TGF-β) (35, 36). William et al. (37) found ZEB1 correlate with an increase in immunosuppressive cell types, including naïve B cells and M2 macrophages within the TME to promote biochemical recurrence. TGF-β is a versatile cytokine that has a dual function in the progression of PCa. Initially, it inhibits cell proliferation and encourages apoptosis; however, in advanced stages, it transitions to a promoter of metastasis by enhancing angiogenesis and EMT (38–40). Wu et al. (41) demonstrated that TGF-β in the TME induces macrophage polarization to the M2 phenotype through the STAT3 pathway. This process, in collaboration with CAFs, leads to increased C-X-C motif chemokine ligand 5 (CXCL5) secretion that further promotes EMT in PCa.

The transition to a mesenchymal phenotype also involves significant alterations in intracellular signaling pathways. The loss of E-cadherin permits the translocation of β-catenin to the cytoplasm, where it participates in the Wnt signaling cascade (42). Nuclear receptor related protein 1(NURR1) promoted oncogenic growth and EMT in PCa by directly transactivating β-catenin, thereby stimulates the Wnt/β-catenin signaling pathway (43). Additionally, Mucin 15 (MUC15), which is downregulated in PCa, represents a potential therapeutic target by inhibiting EMT and cancer stemness through the GSK3β/β-catenin signaling pathway (44). Furthermore, cathepsin K affects the polarization of M2 macrophages in CRPC and regulates tumor progression and metastasis through the IL-17/CTSK/EMT signaling pathway (45).

To successfully metastasize, cancer cells must overcome anoikis—cell death due to detachment—by losing their adhesiveness and acquiring the ability to anchor and proliferate at distant sites (35, 46). This process involves intricate modifications in multiple signaling pathways, including AKT/GSK-3β, Wnt/GSK-3β, ERK, and Notch/NICD3/MMP-3, which collectively facilitate invasion, migration, and eventual distant metastasis of PCa cells (47, 48). Additionally, Tubulin beta 3 (TUBB3) depletion has been shown to reverse anoikis resistance during ECM detachment, inhibiting invasion and migration by significantly reducing activation of the αvβ3/FAK/Src axis—a promising approach for treating bone metastatic PCa (49).

Matrix metalloproteinases (MMPs) are also crucial in PCa progression, as they are highly expressed in cancer cells and facilitate invasion and migration through ECM degradation (50). For instance, MMP-9 derived from osteoclasts has been shown to influence tumor growth within the bone microenvironment by promoting angiogenesis, without affecting the osteolytic or osteogenic changes induced by the tumor (51). Tumor-derived microvesicles can activate fibroblasts, leading to increased motility and resistance to apoptosis via the CX3CL1-CX3CR1 axis (52). MMP-2 and MMP-9 are particularly effective at degrading essential ECM components, including collagen and gelatin. The TR4 nuclear receptor inhibited PCa invasion by modulating macrophage infiltration and the TIMP-1/MMP2/MMP9 axis (53). Additionally, Wang et al. demonstrated that endothelial cells promote metastasis through the IL-6/androgen receptor/TGF-β/MMP-9 signaling pathway in PCa (54). Furthermore, the Notch3-MMP-3 axis has been implicated in osteoblastic lesion formation by inhibiting osteoclast differentiation and promoting osteoblastogenesis (55). Collectively, the interplay between EMT, the TME, and the various signaling pathways is essential in regulating PCa metastasis (**Table 1**).

**Table 1:** Exosome miRNAs associate with prostate cancer metastasis.

Involved process	miRNA	Expression	Targets	Mechanism	Reference
Invasion and migration	miR-888	Upregulated	KLF5, RBL1, TIMP2, SMAD4	miR-888 downregulates KLF5, RBL1, TIMP2, and SMAD4, thereby enhancing invasion and migration	(99)
	miR-214-3p	Downregulated	CCL5	Knocking down AR alters the exosomal circ-DHPS/miR-214-3p/CCL5 pathway, promoting osteoblast recruitment	(100)
	miR-183	Upregulated	TPM1	miR-183 promotes proliferation, invasion, and migration by downregulating TPM1	(101)
EMT	miR-146a-5p	Downregulated	EGFR	miR-146a-5p enhances EMT and accelerates metastasis by modulating the EGFR/ERK pathway	(103)
	miR-1290	Upregulated	GSK3β	miR-1290 from CAFs promotes metastasis via inhibiting GSK3β/β-catenin signaling	(104)
	miR-1246	Downregulated	CDH2, VIM, ZEB1	miR-1246 targets EMT-related genes to inhibit EMT while modulating other cellular processes through the EGFR and PI3K/AKT pathways	(105)
	miR-26a	Downregulated	MMP-2, MMP-9, TIMP-2	miR-26a inhibits proliferation, migration, and metastasis by modulating EMT	(106)
	miR-99b-5p	Downregulated	IGF1R	miR-99b-5p inhibits prostate cancer progression by targeting IGF1R and regulating EMT	(107)
TME	miR-3121-3p	Downregulated	NKX3-1	miR-3121-3p prevents oncogenic dedifferentiation by targeting NKX3-1	(109)
	miR-500a-3p	Upregulated	FBXW7	CAFs exosomes promote metastasis via the miR-500a-3p/FBXW7/HSF1 axis in hypoxic conditions	(110)
	miR-203	Downregulated	pro-inflammatory factors	miR-203 exerted antitumor effect by facilitating M1 macrophage polarization	(111)
	Let-7b-5p	Upregulated	SOCS1	let-7b-5p regulates M2 polarization through the SOCS1/STAT pathway	(113)
	miR-375	Upregulated	DIP2C	miR-375 targets DIP2C, activates the Wnt pathway to promote osteoblastic metastasis	(114)
	miR-940	Upregulated	ARHGAP1 FAM134A	miR-940 enhances the osteogenic differentiation by targeting ARHGAP1 and FAM134A	(115)
Osteoblastic- Osteolytic Bone Metastasis	miR-1275	Upregulated	SIRT2	miR-1275 enhances osteoblast proliferation by modulating SIRT2/Runx2 signaling	(118)
	miR-141-3p	Upregulated	DLC1	miR-141-3p downregulats DLC1, specifically targets osteoblasts	(119)
	miR-214	Downregulated	–	PCa-derived exosomes suppress osteoclast differentiation through the downregulation of miR-214 by inhibiting NF-κB pathway	(121)
	miR-205-5p	Downregulated	RUNX2	Exosomal NEAT1 competitively inhibits miR-205-5p in BMSCs, promoting RUNX2-mediated osteogenesis	(124)
	miR-26a-5p, miR-27a-3p, and miR-30e-5p	Upregulated	BMP-2	miR-26a-5p, miR-27a-3p, and miR-30e-5p collaboratively suppress BMP-2-induced bone formation and osteoblast activity	(126)

CAFs, Cancer-associated fibroblasts; EMT, epithelial-mesenchymal transition; AR, androgen receptor; TME, tumor microenvironment.

### 3.2 Imbalance of TME

PCa cells achieve reactivation and metastatic colonization within the bone microenvironment through complex intercellular interactions and signaling processes. This leads to establish an immunosuppressive environment, which is crucial for evading effective immune responses and forming metastatic foci. Key participants in this process include tumor-associated macrophages (TAMs), T cells, natural killer (NK) cells, and myeloid cells, all of which contribute to bone remodeling and the development of the TME. These cellular components work together to form a niche that supports tumor survival and growth while inhibiting anti-tumor immune responses, thereby facilitating the progression of PCa into bone metastasis.

#### 3.2.1 Tumor-associated macrophages

PCa patients metastasize to bone often exhibit immune abnormalities, including T cell exhaustion and an increased presence of macrophages. TAMs are the predominant immune cell type within the TME and exhibit either pro-inflammatory M1 or anti-inflammatory M2 phenotypes (56). Their presence is significant in prostate bone metastases, where they influence immune responses and tumor behavior (57). Research indicates that targeting TAMs may offer therapeutic benefits in CRPC. For instance, depleting macrophages or inhibiting SRC signaling can alleviate androgen resistance, suggesting new strategies for treatment (58). Utilizing TAMs as anti-tumor effectors or implementing adoptive transfer immunotherapy has emerged as a promising approach (59). Xie et al. (60) found that CircSMARCC1 increases CCL20 secretion via miR-1322 sponging through the CCL20/CCR6 pathway to enhance crosstalk between tumor cells and TAMs. Additionally, Chen et al. (61) demonstrated that the phase separation of the YY1 complex in M2 macrophages promotes IL-6 production, which contributes to tumor progression. Huang et al. (62) demonstrated that TAMs-derived CCL5 promotes the migration, invasion, and EMT of PCa cells, along with the self-renewal of cancer stem cells, by activating the β-catenin/STAT3 pathway. Yu et al. (63) further revealed that endothelial-to-osteoblast transition driven by PCa induces M2 macrophage polarization and immunosuppression in the bone microenvironment via the Wnt pathway.

Innovative therapies are being explored, such as Very Small Size Particles (VSSP), which can polarize macrophages toward the M1 subtype (64). Rydell et al. (65) demonstrated that that VSSP reduces TAMs and inhibits tumor growth in castrated Pten-deficient mice, although it had no effect on Ptenpc-/-; Trp53pc-/- tumors. However, adoptive transfer of VSSP-activated macrophages effectively inhibited tumor growth through reducing angiogenesis and inducing senescence, highlighting the potential of macrophage programming in CRPC therapy. Moreover, the RON receptor tyrosine kinase has been recognized as a key driver in PCa progression by activating M2-polarized macrophages. Camille Sullivan et al. found that loss of RON results in decreased tumor growth and increased macrophage infiltration, promoting M1 marker expression and suppressing M2 markers in PCa (66). Brown et al. (67) discovered that the RON receptor facilitates CRPC progression by recruiting macrophages into the TME, indicating that combining macrophage-targeting agents with RON/Axl inhibition may benefit CRPC patients.

#### 3.2.2 T Cells

T cells, particularly cytotoxic T lymphocytes (CTLs) and helper T cells (Th), are pivotal in the TME of PCa, where they influence both anti-tumor immunity and disease progression (68). CD4+ T cells are crucial for establishing an inflammatory TME; however, they also contribute to immune evasion in PCa (69, 70). Key proteins such as ZAP70 and LAT are essential for T cell activation, and their expression levels can serve as biomarkers to identify metastatic CRPC patients with increased T cell infiltration, potentially guiding immunotherapy approaches (71). Galectin-3 (Gal-3) acts as a significant negative regulator of T cell function, promoting immune resistance by affecting T cell proliferation in lymph nodes and within tumors. Targeting Gal-3 may help to alleviate immune resistance in advanced PCa (72, 73). Furthermore, PTEN-deficient PCa patients show increased levels of FoxP3+ regulatory T cells (Tregs), particularly in metastatic disease, where the ratio of Tregs to CD8+ T cells is skewed. This imbalance fosters an immunosuppressive microenvironment conducive to tumor progression (74). Zhu et al. (75) identified Pygopus 2 (PYGO2) as an oncogene related with poor outcomes of PCa. Their findings indicated that deletion of PYGO2 led to enhanced CTL activation and tumor cell sensitivity to T cell-mediated killing, indicating that PYGO2 contributes to a microenvironment that suppresses immune responses through a p53/Sp1/Kit/Ido1 signaling network. Additionally, Danna et al. (76) demonstrated that un-activated tumor-infiltrating lymphocytes promote osteoclastogenesis in bone metastases, worsening disease progression. In contrast, activated T cells can inhibit osteoclast formation, supporting the rationale for immunotherapy aimed at activating T cells to enhance both anti-cancer and anti-osteoclastic effects.

While T cells generally exert anti-tumor effects, their interaction with the CCL20-CCR6 signaling axis can induce T cell exhaustion, further complicating the immune landscape (77). The presence of functional Tregs reinforces an immunosuppressive niche, promoting bone deposition (78). Notably, the overexpression of basic helix-loop-helix family member e22 (BHLHE22) recruits protein arginase methyltransferase 5 (PRMT5) to enhance colony stimulating factor 2 (CSF2) expression, result in increasing immature neutrophils and monocytes that suppress CD4+ and CD8+ T cell activity, thus contributing to an immunosuppressive bone microenvironment (79).

#### 3.2.3 NK Cells

Natural killer (NK) cells are crucial elements of the innate immune system, providing a primary defense against tumor development (80). In metastatic CRPC, an increase in CD56+ NK cells has been observed following ADT, with higher levels of activated NK cells associated with improved patient outcomes (81, 82). However, resistance to NK cell-mediated responses poses a significant challenge. Recent study indicated that adipocytes in the TME can modulate the effectiveness of NK cells by regulating PD-L1 and natural killer group 2D (NKG2D) expression, with decreased levels of leptin and IL-6 potentially enhancing NK cell activity against CRPC (83). Additionally, bipolar androgen therapy (BAT) has shown promise in treating CRPC by cycling testosterone levels, but high concentrations of dihydrotestosterone (DHT) can suppress NK cell cytotoxicity through androgen receptor pathways, allowing tumor cells to escape immune surveillance. Targeting PD-L1 may restore sensitivity to DHT in CRPC, thus enhancing NK cell function (84).

The infiltrating NK cells within prostate tissues often display an immature yet activated phenotype with diminished cytotoxic potential. Christine et al. (85) found that TGFβ1, secreted in high levels within the prostate environment, contributes to this immunosuppressive effect, especially following cancer cell infiltration. PCa cells further exacerbate this issue by inducing inhibitory receptors such as ILT2/LILRB1 on NK cells and downregulating activating receptors like NKp46, NKG2D, and CD16. This dual mechanism of receptor modulation severely hampers NK cells recognition and elimination of tumor cells. While NK cells are vital for anti-tumor immunity, their effectiveness in PCa is compromised by the TME, which fosters an immunosuppressive environment.

#### 3.2.4 Myeloid cells

Myeloid-derived suppressor cells (MDSCs) are a diverse population of immature myeloid lineage cells that are essential for creating an immunosuppressive environment within the PCa TME (86, 87). These cells are involved in tumor invasion, migration, progression, and metastasis. For instance, Jeong et al. (88) identified that the odorant-binding protein (OBP2A), released from tumors during ADT, captures survival factors like CXCL15 and IL-8. This mechanism not only supports the androgen-independent growth of PCa cells but also enhances MDSC infiltration. Notably, inhibiting OBP2A has been shown to significantly reduce the progression of CRPC and improve the effectiveness of immunotherapies targeting CTLA-4 and PD-1. Furthermore, studies have shown that castration-induced IL-8 promotes the recruitment of polymorphonuclear MDSCs, which further facilitate PCa progression via the IL-8/CXCR2 signaling axis (89). The role of AT-rich interaction domain 1A (ARID1A), a component of the SWI/SNF chromatin remodeling complex, is also crucial; its loss, triggered by inflammation-induced IKKβ activation, leads to increased MDSC chemotaxis and enhances tumor progression through the IKKβ/ARID1A/NF-κB feedback loop (90). Significant modifications in immune modulation and metastatic mechanisms were observed in hybrid cells, characterized by enhanced expression of genes involved in cell adhesion, growth, and cycle progression. Single-cell RNA sequencing revealed an enrichment of tumor-associated neutrophils, monocytes, and macrophages within this hybrid population, indicating an enhanced immunosuppressive capacity (91).

### 3.3 Formation of bone metastasis niche (imbalance of osteoblast-osteoclast)

Before primary tumor cells arrive at distant organs, they secrete regulatory factors that remodel the microenvironment of these distant organs, creating a pre-metastatic niche favorable for tumor colonization and the survival of circulating tumor cells (CTCs) (92). Several hypotheses explain the organotropic mechanisms underlying metastatic spread. Paget’s “seed and soil” theory highlights the crucial role of the target organ’s microenvironment in determining metastatic tropism. In the context of bone, PCa metastasis typically presents as increased osteoblast activity coupled with decreased osteoclast activity, resulting in enhanced bone deposition but compromised bone quality. Vascular cell adhesion molecule 1 (VCAM-1) has been shown to activate dormant micrometastases by recruiting osteoclast progenitor cells (93). Pharmacological inhibition of osteoclast-mediated bone resorption has been found to decrease tumor burden in bone metastases, emphasizing the vital role of osteoclasts in reactivating dormant tumor cells (94).

Activated PCa cells reshape bone tissue to create osteoblastic-dominant metastatic lesions, where osteoblasts, osteoclasts, and immune cells play essential roles in bone remodeling and establishing the metastatic tumor microenvironment (95). The OPG-RANKL-RANK axis mediates a vicious cycle central to this process: Tumor-secreted IL-6 and parathyroid hormone-related peptide stimulate osteoblasts to produce RANKL, which binds to RANK on osteoclasts, initiating bone resorption and releasing growth factors such as TGF-β that support tumor growth (9). The formation of osteoblastic lesions is primarily attributed to PCa-induced inhibition of the Wnt pathway via Dickkopf-1(DKK1) during the early stages of bone metastasis, resulting in a shift from osteolytic to osteogenic dominance. Furthermore, tumor-derived Wnt ligands, BMPs, endothelin-1, FGFs, and IGFs activate osteoblasts at the bone marrow interface, promoting the differentiation of osteoprogenitors and pathological osteogenesis. Although PCa bone metastases are primarily osteoblastic, emerging evidence underscores the critical role of osteoclasts. A recent study demonstrated that exosomes derived from PCa cells are vital mediators of bone homeostasis, facilitating osteoclastogenesis and inhibiting osteoblast differentiation both *in vitro* and *in vivo*, thus establishing an osteolytic pre-metastatic niche (96). These findings enhance our understanding of the molecular mechanisms by which osteoclasts are involved in bone metastasis and identify potential protein biomarkers for monitoring disease progression and therapeutic efficacy in PCa bone metastasis.

In addition to exosomes secreted by tumor cells, stromal cells also release exosomes into the tumor microenvironment, thereby influencing tumor metabolism. The metabolic state of tumor cells differs from that of normal cells, and exosomes derived from CAFs have been shown to regulate metabolic reprogramming in PCa cells. Specifically, these exosomes inhibit mitochondrial oxidative phosphorylation (OXPHOS), enhance glycolytic activity, and reduce the pH of the tumor microenvironment. These changes support a metabolic state that favors tumor survival and facilitate the adaptation of PCa cells to hypoxic conditions (97).

## 4 Molecular mechanism of exosome miRNA regulating TME

Accumulating evidence suggests that exosome-derived miRNAs serve as a novel information transfer system within the organism, enhancing communication between cells and tissues during tumor development. These bioactive molecules are delivered to recipient cells, where they promote tumor progression by affecting invasion, migration, EMT, and the regulation of the TME. Consequently, understanding the mechanisms through which exosome miRNAs influence the bone marrow microenvironment may offer valuable insights for developing targeted therapeutic strategies to mitigate PCa metastasis.

### 4.1 Intrinsic regulation of tumor cells (invasion and migration, EMT)

#### 4.1.1 Invasion and migration

Exosomes miRNAs are significant contributors to facilitating cell invasion and migration (98), two hallmark characteristics of aggressive PCa. These exosomal miRNAs influence various signaling pathways that boost the invasion and migration of tumor cells, thereby aiding in the formation of metastatic niches within the bone marrow. miR-888, has been recognized as a key contributor in promoting PCa cell proliferation and migration. Enriched in PC3-ML cells, exosomal miR-888 downregulates important proteins such as Krüppel-like factor 5 (KLF5), retinoblastoma-like protein 1 (RBL1), tissue Inhibitor of metalloproteinases 2 (TIMP2), and SMAD family member 4 (SMAD4), enhancing overall tumor cell capabilities (99). Furthermore, Yang et al. (100) investigated how exosomes contribute to the migration of PCa cells toward osteoblasts. Their research revealed that exosomes from AR-silenced cancer cells or those exposed to the androgen receptor antagonist enzalutamide elevated the levels of circular RNA-deoxyhypusine synthase (circ-DHPS). Acting as a ceRNA for miR-214-3p, this circ-DHPS triggers increased CCL5 secretion by osteoblasts. Higher concentrations of CCL5 promote the recruitment of more PCa cells to the bone environment. Disrupting the circ-DHPS/miR-214-3p/CCL5 interaction may offer a strategy to reduce the migration of cancer cells. Additionally, Dai et al. (101) reported that high levels of miR-183 enhanced the invasion and migration by downregulating tropomyosin 1 (TPM1) in PCa cells.

#### 4.1.2 EMT

Exosomal miRNAs are critical regulators of EMT in PCa, significantly impacting tumor progression and metastasis. Studies have shown that various PCa cell populations release exosomes containing over 1,800 distinct miRNAs, which can alter the local tumor microenvironment and promote cancerous behaviors (102). The influence of exosomal miRNAs extends beyond PCa cells to include contributions from various cell types, such as CAFs and TAMs. CAFs release exosomes containing miR-146a-5p, which PCa cells (LNCaP and DU145) subsequently internalize, inhibiting the EGFR/ERK signaling pathway and thereby promoting EMT and metastasis (103). Similarly, exosomal miR-1290 derived from CAFs facilitates cancer cell proliferation and metastasis through the suppression of the GSK3β/β-catenin pathway (104). Another interesting aspect is the selective secretion of miR-1246 from PCa cells, which inhibit EMT through the EGFR and PI3K/AKT pathways (105). Exosomal miR-26a from low-grade prostate carcinoma cells (LNCAP) suppresses the malignant behaviors of metastatic CRPC cells (PC-3), indicating that miR-26a may play a regulatory role in tumor growth and metastasis by altering expressions of EMT-related factors (106). Interestingly, in PCa tissues and cell lines, miR-99b-5p levels are reduced, whereas they are heightened in exosomes from human bone marrow mesenchymal stem cells (HBMSCs). These exosomes from HBMSCs diminish the malignant traits of PCa cells, with mimics of miR-99b-5p further amplifying this inhibitory effect. Conversely, inhibiting miR-99b-5p promoted PCa progression *in vitro*. Mechanistically, miR-99b-5p inhibits cancer progression by targeting insulin-like growth factor 1 receptor (IGF1R) and regulating EMT (107).

### 4.2 Immune cells interaction

Exosomes miRNAs are crucial in modifying the TME in PCa, primarily through the transfer of miRNAs that facilitate intercellular communication. CAFs, integral components of the stroma, significantly contribute to tumor growth through mechanisms often driven by TGF-β (108). Chise et al. (109) showed that miR-3121-3p derived from CAFs can suppress the oncogenic dedifferentiation of PCa cells by targeting NK3 Homeobox 1 (NKX3-1), especially in androgen-sensitive and AR-dependent environments. Hypoxia, a common feature in primary metastatic lesions, further complicates the TME. Liu et al. (110) found that CAFs under hypoxic conditions secrete exosomes enriched with miR-500a-3p, which significantly promote PCa metastasis. This miRNA targets F-box and WD repeat domain-containing 7 (FBXW7), indicating that CAF exosomes drive metastasis via the miR-500a-3p/FBXW7/HSF1 pathway. This suggests that targeting hypoxia or exosomal miR-500a-3p could represent effective strategies for managing advanced PCa. Exosomal miRNAs also influence immune cell behavior within the TME. Notably, miR-203, found in exosomes from PCa cells, can induce M0 macrophages to polarize toward the anti-tumor M1 phenotype, thereby inhibiting proliferation, migration, and invasion while promoting apoptosis in LNCAP cells. *In vivo* studies further support the promise of miR-203 as a therapeutic target, as its upregulation correlates with reduced tumor growth and increased M1 macrophage markers within the TME (111).

Moreover, exosomal miRNAs can induce significant phenotypic transitions within the immune system. let-7b derived from PC-3 cells can be transferred to THP-1 monocytes, leading to polarization into a TAMs-like phenotype that promotes tumor growth (112). Specifically, let-7b-5p reduced the levels of suppressor of cytokine signaling 1 (SOCS1) and enhances the phosphorylation of signal transducer and activator of transcription 1/3/5 (STAT1, STAT3, and STAT5), suppressing macrophage phagocytic activity. Inhibition of let-7b-5p reverses these effects, thereby enhancing macrophage function and decreasing the proliferation of PCa cells (113). The relationship between PCa and bone cells is also significant in understanding the TME. PCa metastasis often leads to both osteoblastic and osteolytic bone metastases, with tumor cells interacting with osteoblasts, osteoclasts, and mesenchymal stem cells. Liu et al. (114) discovered that miR-375 is markedly elevated in advanced PCa as well as in metastatic cell lines. This microRNA not only fosters osteoblastic differentiation in human mesenchymal stem cells (hMSCs) but also boosts the proliferation and invasion of PCa cells. At a mechanistic level, miR-375 directly targets disco-iinteracting protein 2 homolog C (DIP2C), which results in the activation of the Wnt signaling pathway and promotes osteoblastic differentiation in hMSCs. Furthermore, exosomal miR-940 derived from C4-2B cells enhances the osteogenic differentiation of hMSCs by targeting Rho GTPase activating protein 1 (ARHGAP1) and family with sequence similarity 134 member A (FAM134A) (115).

### 4.3 Formation of pre-metastatic niche

#### 4.3.1 Osteoblastic bone metastasis

Recent studies have underscored the critical role of exosome in the formation of pre-metastatic niches and immune suppression (116). Hoshino et al. demonstrated that tumor-derived exosomes contribute to the establishment of microenvironments that favor metastasis (117). Additionally, primary PCa cells remodel the bone marrow microenvironment into a tumor-friendly niche by secreting exosomes containing miRNAs, lncRNAs, and proteins, thereby facilitating tumor cell colonization and growth.

Osteoblasts, which are pivotal in PCa bone metastasis, are regulated by exosome mechanisms. *In vitro* experiments indicate that PCa-derived exosomes promote the proliferation and activation of human osteoblast cell lines, thereby creating a microenvironment conducive to subsequent tumor cell colonization. The Runt-related transcription factor 2 (RUNX2), a master regulator of osteogenic differentiation, is critically involved in maintaining bone microenvironment homeostasis. Exosomal miR-1275 enhances osteoblast activity by inhibiting the deacetylase Sirtuin 2, which leads to the upregulation of RUNX2 expression and a significant increase in osteogenic markers such as osteocalcin, type I collagen, and osteopontin (118). Exosomal miR-141-3p remodels the pre-metastatic niche by downregulating deleted in liver cancer 1 (DLC1), which encodes DLC1 Rho GTPase-activating protein and specifically targets osteoblasts (119). Furthermore, murine PCa-derived exosomes have been shown to inhibit the differentiation of osteoclast precursors and impair bone formation capacity in metastatic lesions (120).

Bone metastasis involves multifaceted regulatory crosstalk, with exosomes modulating the activity of osteoblasts and other bone marrow cells, including osteoclasts, monocytes, and mesenchymal stem cells (MSCs). Recent *in vitro* studies reveal that PCa-derived exosomes suppress osteoclast differentiation through the downregulation of miR-214 (121). Additionally, exosomes from PCa cells carrying integrin αvβ6 promote M2 monocyte polarization and facilitate osteogenic metastasis (122). Human bone marrow-derived MSCs (BMSCs), which are multipotent progenitors capable of differentiating into osteoblasts, chondrocytes, and adipocytes (123), are increasingly recognized as targets of exosome osteogenic reprogramming. Mo et al. reported that the exosome-transported nuclear-enriched abundant transcript 1 (NEAT1) is delivered to BMSCs, where it competitively binds to miR-205-5p, leading to RUNX2 upregulation and increased osteogenic protein expression in the PCa bone microenvironment (124).

#### 4.3.2 Osteolytic bone metastasis

PCa-derived exosomes inhibit osteoclast function. In addition to osteoclast hyperactivation driving osteolytic lesions, recent studies have revealed a novel mechanism whereby exosomal miR-92a-1-5p from osteoblastic, osteoclastic, or mixed PCa subtypes promotes osteoclast differentiation while suppressing osteogenesis through the targeting of COL1A1, which encodes collagen type I alpha 1 chain, a major component of the bone ECM. The bone-destructive potential of these PCa subtypes correlates with the levels of osteogenic miRNAs (miR-148a-3p, miR-375) and osteoclastic miR-92a-1-5p (96). Moreover, PCa exosome containing miR-26a-5p, miR-27a-3p, and miR-30e-5p collaboratively suppress BMP-2-induced bone formation and osteoblast activity (125).

## 5 Clinical applications and challenges

### 5.1 Diagnostic biomarkers

Early detection and treatment of PCa are crucial for improving patient prognosis and long-term survival. Clinically, the Gleason score exhibits strong correlations with the biological behavior of PCa. Bertoli et al. (126) demonstrated that miR-153 is upregulated in PCa patients with high Gleason scores and plays a critical role in regulating PCa cell proliferation, migration, and invasion. Notably, miR-153 is secreted by exosomes within the tumor microenvironment, and its release into peritumoral tissues significantly influences tumor cell growth. Exosomal miRNAs show promise for predicting aggressive or localized metastasis, thereby aiding in the differentiation between normal tissues, benign prostatic hyperplasia (BPH), and aggressive PCa. Duca et al. (127) reported that hsa-miR-19b-3p and hsa-miR-101-3p are significantly elevated in the blood of PCa patients compared to healthy controls. Furthermore, these miRNAs are markedly increased in prostate tumor tissues relative to normal adjacent tissues (NATs). Receiver operating characteristic (ROC) analysis revealed that hsa-miR-19b-3p effectively discriminates tumor tissues from NATs, while hsa-miR-101-3p distinguishes metastatic from non-metastatic PCa patients. Additionally, studies indicate that miR-2909 is upregulated in urinary exosomes of PCa patients compared to healthy individuals and promotes tumor cell invasion (128). Collectively, these findings suggest that exosomal miRNAs in the biofluids of PCa patients have the potential to serve as robust diagnostic biomarkers and may be valuable tools for guiding prostate biopsy decisions.

### 5.2 Targeted therapy strategy

Current therapeutic strategies for PCa encompass endocrine therapy, chemotherapy, radiotherapy, and surgery. While these treatments can significantly delay or suppress disease progression, chemoresistance frequently leads to patient mortality. Shan et al. (129) demonstrated that exosomes derived from CAFs reduce the chemosensitivity of PCa cells, enhancing drug resistance in resistant subpopulations. Specifically, exosomes from PCa-associated CAFs carrying miR-423-5p increase resistance to taxanes by suppressing GREM2 through the TGF-β pathway, thereby amplifying PCa cell sensitivity to these agents *in vivo*. ADT, which is the cornerstone of advanced PCa treatment, is often compromised by adaptive mechanisms. The therapeutic management of CRPC is particularly challenging due to resistance to docetaxel and other agents. Studies on miR-34a modulation in PCa cells reveal its regulatory effect on B-cell lymphoma 2 (BCL-2), partially influencing cancer cell responses to docetaxel (130). Gan et al. (131) found that miR-375 directly disrupts the expression of phosphatase non-receptor type 4 (PTPN4), stabilizing phosphorylated STAT3. Zhou et al. (132) observed a significant upregulation of miR-217 and downregulation of miR-23b-3p in plasma exosomes from PCa patients compared to healthy controls; both miRNAs are potentially involved in modulating tumor cell proliferation and invasion. TAMs play a pivotal role in intercellular communication within the tumor microenvironment. Guan et al. (133) analyzed miRNA profiles in exosomes released by THP-1 and M2 macrophages, revealing elevated levels of miR-95 in TAM-derived exosomes, which are directly internalized by recipient PCa cells. *In vitro* and *in vivo* loss-of-function assays demonstrated that miR-95 functions as an oncogenic driver by binding to its downstream target JunB, promoting PCa cell proliferation, invasion, and EMT.

### 5.3 Clinical translational challenges

#### 5.3.1 Complexity in target selection and validation

The primary challenge in exosomal miRNA-based therapies is the selection and validation of appropriate targets. Exosome miRNAs have the capacity to simultaneously regulate multiple genes, complicating the elucidation of their specific mechanistic roles. Although advanced algorithms, extensive sequencing data, and databases such as MiRBase facilitate the prediction of miRNA-mRNA binding sites, the functional annotations for most miRNAs remain incomplete, and their biological contexts are often inadequately validated.

In clinical applications, exosome miRNA mimics are primarily used to restore deficient or underexpressed miRNAs. In this context, target specificity is less critical than identifying miRNAs that can directly modulate disease-relevant pathways. However, the inherent heterogeneity of miRNA expression in diseases such as cancer—further influenced by TME—poses significant challenges in identifying a universally effective miRNA therapeutic across diverse tumor types (134).

#### 5.3.2 Off-target effects and safety concerns

The pleiotropic nature of exosome miRNAs raises concerns about off-target effects, wherein unintended suppression or activation of non-target genes may lead to adverse outcomes. For example, anti-miRNA therapies may inadvertently target tumor suppressor genes or genes essential for cellular homeostasis, thereby disrupting critical physiological functions. Additionally, the potential for cross-interactions between therapeutic miRNAs and other ncRNAs complicates clinical translation (135).

#### 5.3.3 Delivery challenges and toxicity

A major bottleneck for exosome miRNA therapeutics, similar to other RNA-based modalities, is delivery efficiency and specificity. Ideal delivery vehicles must achieve robust tissue- or cell-specific targeting while avoiding immune activation or cytotoxicity. Although lipid nanoparticles (LNPs) and other systems have demonstrated success in delivering siRNA and mRNA vaccines, miRNA delivery remains suboptimal. Early clinical trials using LNPs for miRNA delivery have yielded limited efficacy, prompting ongoing research into tumor-targeted delivery systems (136). Current strategies focus on the precision engineering of carriers to enhance bioavailability while minimizing off-tissue accumulation (137).

## 6 Conclusion and Perspectives

In the therapeutic landscape of PCa, radiotherapy has gained increasing prominence alongside traditional castration therapy and chemotherapy. Strategies that focus on modulating TME show significant promise. Notably, genetic engineering modifications of exosome miRNA can enhance radiosensitization to reverse tumor radioresistance, while engineered exosome miRNA loaded with radionuclide transport proteins improve the precision and efficacy of internal radiation therapy (138). In drug delivery, ligand-specific engineered, nanoparticle-based delivery systems or chemical modifications exosome miRNA facilitates site-specific targeting, enhancing drug bioavailability through improved tissue permeability while minimizing systemic toxicity (139). Immunologically, although tumor-derived exosomes carrying antigens contribute to immune evasion, innovative technologies such as γ-interferon-modified exosome vaccines can reprogram immune responses by suppressing key angiogenesis-related proteins, thereby inhibiting metastatic lesions (140). When combined with tumor cell vaccines, this approach effectively dual-blocks immunosuppressive signaling, amplifying antitumor effects. Future research must advance mechanistic exploration and clinical translation in parallel. This includes vertically dissecting the exosomal miRNA-target gene-signaling pathway regulatory network and horizontally establishing dynamic interaction models that encompass TME components. These models should be validated through clinical trials to assess their multifaceted roles as prognostic biomarkers, therapeutic response monitors, and novel therapeutic targets.

**Figure 1:**
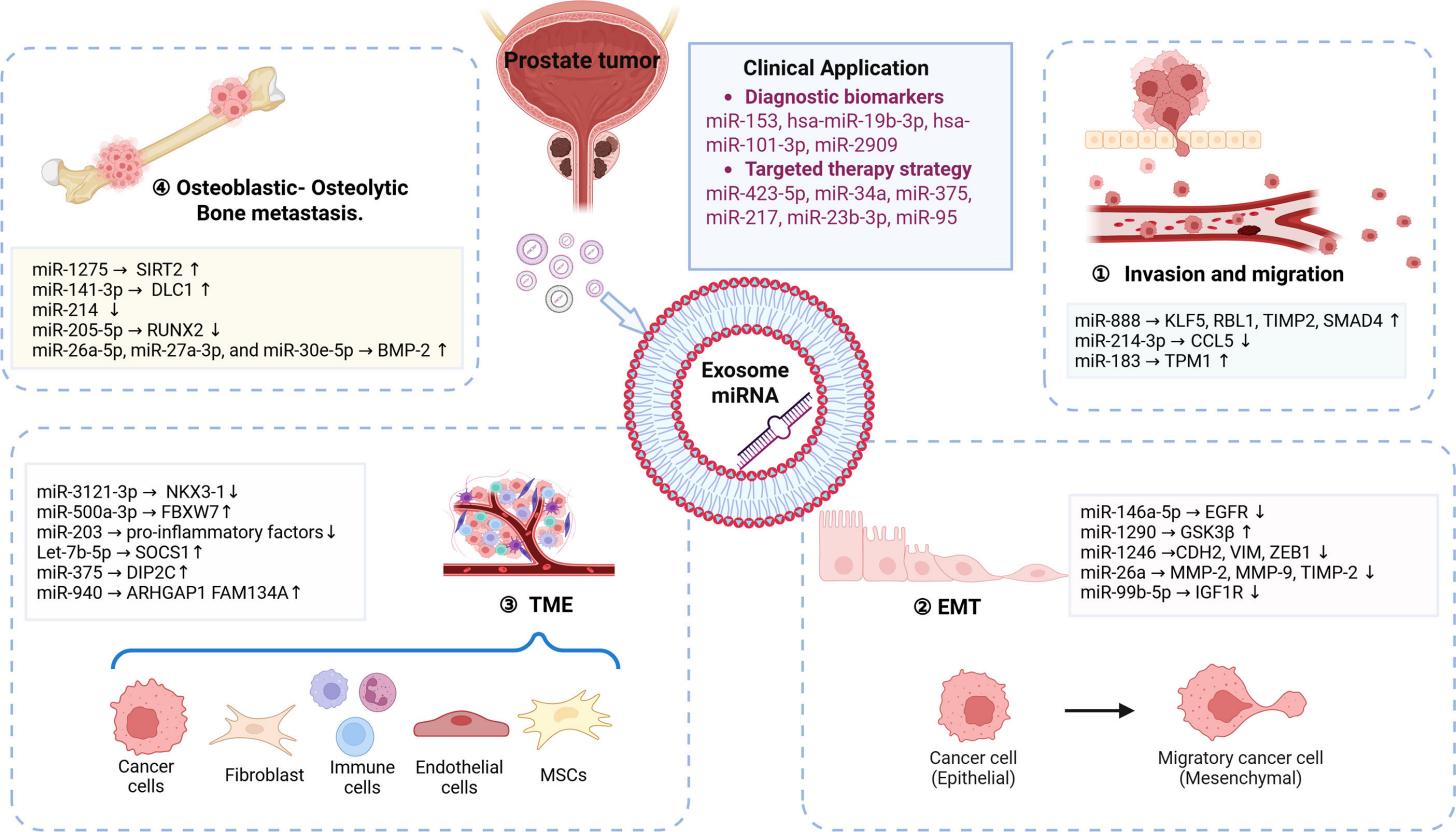
Exosomal miRNAs mediate prostate cancer metastasis.

Exosomal miRNAs play a central role in PCa progression and metastasis via TME modulation and intercellular communication. This review systematically consolidates the multifaceted regulatory mechanisms of exosomal miRNAs in prostate cancer metastasis, innovatively proposing a theoretical framework in which these miRNAs synergistically drive TME remodeling through a “targeted cascade regulatory network.” (**Figure 1**). It highlights their dual potential for clinical translation as both predictive biomarkers for metastasis and therapeutic targets—a “double-edged sword”—with applications in precision interventions. However, current research inadequately characterizes the spatiotemporal dynamics of the native TME in patients. Challenges remain regarding the specificity of exosomal miRNAs, including the heterogeneity of signals derived from tumor versus non-tumor sources. Additionally, gaps in technical standardization related to isolation and detection methods, as well as a disconnect in the clinical translation data from phases II and III trials, hinder progress. Furthermore, clinical translation faces challenges, including off-target effects, delivery hurdles, and safety concerns. To bridge the gap between research and clinical application, collaborative innovation systems that integrate basic research with clinical oncology are critical. Leveraging multi-omics data and novel platforms will be essential for developing precision therapies that improve outcomes in advanced PCa.
